# Anesthetic Management of an Adult Patient Post a Fontan Procedure in Laparoscopic Surgery: A Case Report

**DOI:** 10.7759/cureus.59594

**Published:** 2024-05-03

**Authors:** Sho Takemoto, Masako Asada, Jun Maki, Hidekazu Setoguchi, Sumio Hoka

**Affiliations:** 1 Center for Transplantation Sciences, Department of Surgery, Massachusetts General Hospital and Harvard Medical School, Boston, USA; 2 Department of Cardiovascular Surgery, Kyushu University Graduate School of Medical Sciences, Fukuoka, JPN; 3 Department of Anesthesiology and Critical Care Medicine, Kyushu University Graduate School of Medicine, Fukuoka, JPN; 4 Intensive Care Unit, Kyushu University Hospital, Fukuoka, JPN; 5 Department of Anaesthesiology, Kyushu University Beppu Hospital, Oita, JPN

**Keywords:** anesthetic management, non-cardiac surgery for adult congenital heart disease patient, laparoscopic surgery, general anesthesia practice, fontan physiology

## Abstract

We report the successful anesthetic management of laparoscopic surgery in a 21-year-old female patient with Fontan circulation. A preoperative careful review of cardiac catheterization results helped assess the risk of the surgery and implement anesthetic management. Intraoperative management focused on minimizing the impact on pulmonary vascular resistance and venous return by optimizing ventilation and applying lower pneumoperitoneum pressure without tilting the position. Milrinone was administered to reduce pulmonary vascular resistance and provide inotropic support with minimally invasive monitoring. The patient remained stable throughout the procedure without complications. This case highlights the importance of thorough preoperative assessment, individualized intraoperative management, and collaboration with the surgical team when caring for adult Fontan patients undergoing laparoscopic surgery.

## Introduction

Fontan circulation, a palliative surgical approach for complex congenital heart disease resulting in single-ventricle physiology, is based on a delicate balance of preloaded volume, pulmonary vascular resistance (PVR), single ventricular function, and systemic vascular resistance. Special consideration is required during general anesthesia for laparoscopic surgery in patients with Fontan circulation because pneumoperitoneum with carbon dioxide (CO_2_) gas and the Trendelenburg or reverse Trendelenburg position could significantly affect Fontan physiology.

As improvements in clinical outcomes enable more children with Fontan circulation to survive into adulthood, the number of non-cardiac surgeries for adult patients, including laparoscopic surgery, is increasing [1]. While numerous review articles have discussed anesthetic management strategies for patients at various stages of palliation, the unique challenges posed by laparoscopic surgery in adult Fontan patients warrant individualized approaches. Herein, we describe the anesthetic management of an adult female patient with Fontan circulation who successfully underwent laparoscopic surgery.

## Case presentation

The patient was a 21-year-old female with a body weight of 52 kg and a body mass index of 22 who presented with right ovarian cysts and was scheduled for laparoscopic ovarian cystectomy. She had been diagnosed with mitral atresia and had undergone the Glenn procedure at the age of two years and the Fontan procedure at the age of four years. She had well-maintained Fontan circulation and no physical sign of hypoxemia or venous congestion. Regular medications were warfarin, aspirin, and candesartan. She was classified according to the American Society of Anesthesiologists Physical Status Classification System (ASA-PS) as 3, based on the complex cardiac history and the presence of single-ventricle physiology. The preoperative vitals for standard ASA monitoring were as follows: blood pressure: 100/66 mmHg, heart rate: 76 beats per minute with normal sinus rhythm, respiratory rate: 10 breaths per minute, oxygen saturation: 95% on room air, and body temperature: 36.6°C. The last cardiac catheterization at the age of 19 years showed a cardiac index of 2.8 L/min/m^2^, arterial oxygen saturation of 95%, Qp/Qs of 1.0, and Rp/Rs of 1.1/25.2. Venous pressure at the inferior vena cava, superior vena cava, and the extra-cardiac conduit were the same at 11 mmHg. The right and left pulmonary artery pressures were 10 mmHg, and the right and left pulmonary artery wedge pressure were 7 mmHg. The preoperative echocardiogram showed a single-ventricular ejection fraction of 71%. There was no significant local asynergy on the single-ventricle, valve dysfunction, or pressure gradient at the conduit anastomosis sites.

**Figure 1:**
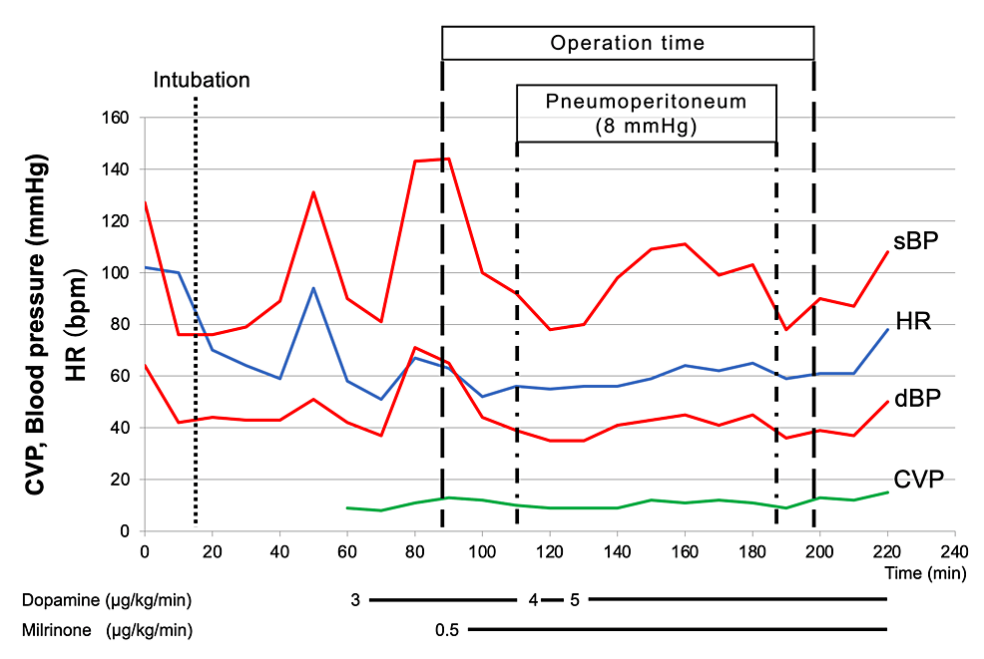
Anesthesia record HR, heart rate; sBP, systolic blood pressure; dBP, diastolic blood pressure; CVP, central venous pressure

**Figure 2:**
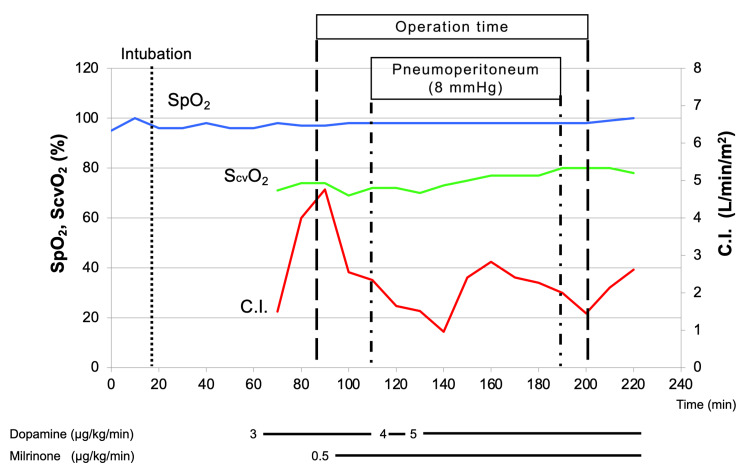
Anesthesia record SpO_2_, peripheral capillary oxygen saturation; ScvO_2_, central venous oxygen saturation; C.I., cardiac index

The patient was placed on the standard spine position, and a peripheral intravenous and a radial artery line were inserted. General anesthesia was induced with 1-2% sevoflurane, 90 mg of propofol, 100 µg of fentanyl, and 30 mg of rocuronium under TOF-Watch^TM^ (Organon, Swords Co., Dublin, Ireland) monitoring. The patient was intubated uneventfully after the administration of lidocaine spray on the larynx. Anesthetic maintenance consisted of adjusting concentrations of sevoflurane in air/oxygen and the dose of remifentanil. Pressure-controlled ventilation was applied, and a peak inspiratory pressure of 16 cmH_2_O was required to maintain a tidal volume of 7 mL/kg. Positive end-expiratory pressure (PEEP) was set at 4 cmH_2_O. Mild hyperventilation with a respiratory rate of 12 to 14/min was maintained, targeting arterial partial pressure of CO_2_ (PaCO_2_) of 32 to 35 mmHg. We monitored central venous pressure (CVP), central venous oxygen saturation (ScvO_2_), cardiac index, and stroke volume variety (SVV) using a FloTrac^TM^ sensor (Edwards Lifesciences, Irvine, CA, USA) and PreSep^TM^ oximetry catheter (Edwards Lifesciences). Intravenous milrinone was administered continuously at 0.5 µg/kg/min during surgery. The flat spine position was maintained, and the intra-abdominal pressure (IAP) was set at 8 mmHg to prevent venous return impairment. Blood pressure was decreased by general anesthesia, positive airway pressure ventilation, and increased IAP but could be recovered immediately by meticulous volume loading under SVV monitoring and intravenous dopamine and phenylephrine support. CVP, oxygen saturation, and ScvO_2_ were stable through the surgery (Figures 1, 2). The procedure was completed uneventfully. Operation time was 109 minutes, and general anesthesia time was 219 minutes. The patient was extubated in the operating room and taken to the recovery room. There was no postoperative surgical or anesthetic complication.

## Discussion

Adult patients with Fontan circulation show broad-spectrum clinical status. The key mechanism is that increased contractility alone does not increase cardiac output at rest in Fontan patients [2]. Pulmonary blood flow depends on CVP, PVR, functional left atrial pressure, and single ventricular end-diastolic pressure. Maintaining appropriate CVP, lowering PVR and single ventricular end-diastolic pressure, and optimizing systemic blood pressure, which acts as an afterload, are essential for maintaining Fontan circulation [2]. In this case, we used milrinone, a phosphodiesterase III inhibitor that reduces afterload and PVR, as a primary support for Fontan circulation. Inhaled nitric oxide was available as a next-step support but was not required. Given the patient's favorable preoperative single-ventricular ejection fraction, dobutamine, a beta-adrenergic agent, was not used due to its associated risk of tachycardia. Dopamine, which has dose-dependent effects on dopaminergic, beta-adrenergic, and alpha-adrenergic receptors, was chosen to provide inotropic support. At the doses used in this case (3-5 mcg/kg/min), dopamine primarily stimulates beta-adrenergic receptors and has a mild alpha-adrenergic effect, which could provide increasing in heart rate, cardiac contractility, output, and systemic vascular resistance. We chose dopamine over dobutamine due to its relatively weaker beta-adrenergic effects at these doses, minimizing the risk of potential tachycardia and arrhythmias associated with dobutamine. Phenylephrine, a selective alpha-1 adrenergic receptor agonist, was used to counteract the vasodilatory effects of general anesthesia and pneumoperitoneum, thereby maintaining systemic vascular resistance and blood pressure. The combination of milrinone, dopamine, and phenylephrine allowed for a balanced approach to supporting Fontan circulation.

Intraoperative invasive monitoring plan should be based on the patient's preoperative Fontan status and surgical risks. While preoperative cardiac catheterization is not always necessary for stable Fontan patients undergoing non-cardiac surgery, reviewing the most recent cardiac catheterization results is highly recommended. In cases where the patient has well-maintained Fontan circulation, and the surgery is expected to be of short duration, invasive monitoring may be unnecessary. However, invasive monitoring should be considered in patients with poor Fontan circulation, those undergoing complex surgical procedures, or when the anesthesiologist has limited experience in managing adult Fontan patients. Combining arterial blood pressure monitoring with the FloTrac^TM^ sensor can be a valuable option. The FloTrac^TM^ system enables continuous monitoring of cardiac output and SVV, which is particularly effective for optimal volume control in Fontan patients with a narrow volume management range. Yoshikawa et al. reported that incorporating hypotension prediction index monitoring alongside FloTrac^TM^ could help reduce the cumulative amount of intraoperative hypotension during major non-cardiac surgery [3]. The PreSep^TM^ oximetry catheter is also an additional option. Continuous monitoring of ScvO_2_ serves as a highly sensitive marker of tissue perfusion, as it accurately reflects the adequacy of tissue oxygenation, which allows for the early recognition of peripheral perfusion issues and interventions. In this case, we experienced blood pressure drops after initiating positive airway pressure ventilation and establishing pneumoperitoneum. However, with our comprehensive monitoring plan, we could effectively and promptly manage this hypotensive episode with optimal vasopressor while minimizing volume load and maintaining euvolemic fluid status. It is also important to be aware of the risk of secondary venous thrombus formation after central venous line placement in Fontan patients while they often take warfarin or aspirin.

Laparoscopic surgery poses additional challenges for patients with Fontan circulation, including CO_2_ pneumoperitoneum and the Trendelenburg or reverse Trendelenburg position. Increased IAP causes diaphragm elevation, which results in increased airway pressure and decreased pulmonary compliance, and these changes lead to hypoventilation. The collapse of basal lung tissue, decreased functional residual capacity, ventilation-perfusion ratio (V/Q) mismatch, and increased intrapulmonary shunting, all lead to hypoxemia [4]. Hypoventilation and CO_2_ gas absorption via the peritoneum increase the PaCO_2_ level, which could have a significant impact on PVR. While these issues can be addressed by applying PEEP or increasing peak inspiratory pressure, it is crucial to consider that all these changes and increased airway pressure could be detrimental to Fontan circulation. During laparoscopic surgery, a PEEP of 5 cmH_2_O is considered essential to decrease intraoperative atelectasis due to pneumoperitoneum [4]. We applied minimal PEEP with pressure-controlled ventilation to mitigate the risk of increasing airway pressure. Additionally, we controlled PaCO_2_ at around the lower normal limit range with mild hyperventilation to minimize the impact on PVR. It should be noted that even mild hyperventilation can adversely affect the Fontan circulation since hyperventilation itself can increase airway pressure. Aksakal et al. reported that 8 mmHg insufflation increased PaCO_2_ from a mean value of 33.4 mmHg to 35 mmHg and, more interestingly, to 38.1 mmHg after the end of insufflation in adult patients without heart disease undergoing laparoscopic cholecystectomy [5]. They suggested IAP might suppress CO_2_ absorption from the capillaries. Careful attention should be paid to changes in blood gases and hemodynamics not only during insufflation but also after its completion.

Furthermore, hypercarbia and reduced venous return caused by IAP elevate sympathetic nerve activity, which results in tachycardia and decreasing cardiac output [3]. Oiwa et al. reported that sympathetic dominance occurs in a pneumoperitoneum duration-dependent manner, with increased baroreceptor sensitivity and risk of hemodynamic changes in the head-down position [6]. Several reports described successful laparoscopic surgery for adult Fontan patients using IAP < 10 mmHg [7,8]. In our case, we applied a flat spine position and limited pneumoperitoneum pressure based on a thorough preoperative discussion with the surgical team. Close collaboration between anesthesiologists and surgeons is essential as limited IAP and flat spine position may present technical challenges for laparoscopic surgery.

## Conclusions

We report the successful anesthetic management of laparoscopic surgery in an adult patient with Fontan circulation. Careful preoperative assessment, intraoperative monitoring, and individualized management tailored to Fontan physiology are crucial. Close collaboration with surgeons regarding factors that can affect technical challenges is also essential for ensuring optimal outcomes.

## References

[1] McNamara JR, McMahon A, Griffin M (2022). Perioperative management of the Fontan patient for cardiac and noncardiac surgery. J Cardiothorac Vasc Anesth.

[2] Gewillig M, Brown SC (2016). The Fontan circulation after 45 years: update in physiology. Heart.

[3] Yoshikawa Y, Maeda M, Kunigo T (2024). Effect of using hypotension prediction index versus conventional goal-directed haemodynamic management to reduce intraoperative hypotension in non-cardiac surgery: a randomised controlled trial. J Clin Anesth.

[4] Srivastava A, Niranjan A (2010). Secrets of safe laparoscopic surgery: anaesthetic and surgical considerations. J Minim Access Surg.

[5] Aksakal N, Taviloglu K, Yanar HT (2017). The effects of pneumoperitoneum pressure on blood gases, respiratory and venous systems during laparoscopic cholecystectomy: a prospective randomized trial. Laparosc Endosc Surg Sci.

[6] Oiwa A, Terada T, Ochiai R (2016). Reconsidering minimally invasive surgery: a quantitative analysis of nerve function in gynecological laparoscopic surgery [Article in Japanese]. J Jpn Soc Clin Anesth.

[7] Pans SJ, van Kimmenade RR, Ruurda JP, Meijboom FJ, Sieswerda GT, van Zaane B (2015). Haemodynamics in a patient with Fontan physiology undergoing laparoscopic cholecystectomy. Neth Heart J.

[8] McClain CD, McGowan FX, Kovatsis PG (2006). Laparoscopic surgery in a patient with Fontan physiology. Anesth Analg.

